# Supplementary material for: Expression Quantitative Trait Methylation Analysis Identifies Whole Blood Molecular Footprint in Fetal Alcohol Spectrum Disorder (FASD)
Source: Int J Mol Sci. 2023 Apr 1;24(7):6601. doi: 10.3390/ijms24076601 (PMC10095438; doi:10.3390/ijms24076601)
Supplement: Supplementary file 1 [file ijms-24-06601-s001.zip › FASD_SupplementaryFile1_130223.docx]

set.seed(123)

library(doParallel)

registerDoParallel(cores = 16)

##Read raw EPIC data

library(minfi)

targets = read.metharray.sheet("~/lpersonal/FASD/DNA/idats", pattern = "pheno_extremeDNA.csv")

rgSet = read.metharray.exp(targets=targets)

##Methylaid quality control for detecting outliers

library(MethylAid)

Methylaid.data = summarize(targets, file ="~/lpersonal/FASD/DNA/preprocessNoob_extreme/Methylaid-report")

visualize(Methylaid.data,thresholds=list(hm450k=list(MU=10.5, OP = 12, BS = 12, HC = 13, DP = 0.95)))

##EPIC analysis

annotation = getAnnotation(rgSet)

annotation.gr = makeGRangesFromDataFrame(annotation, keep.extra.columns = T, seqnames.field = "chr", strand.field = "strand", start.field = "pos", end.field = "pos")

GMset = preprocessNoob(rgSet)

library(Hmisc)

autosomes = annotation[!annotation$chr %in% c("chrX","chrY"), ]

GMset.noXY = GMset[featureNames(GMset) %in% row.names(autosomes),] #Remove XY

popprobes = read.csv("~/lpersonal/FASD/DNA/annotation/Polymorphic-CpGs-Illumina450k.csv", sep=",",header=T)

popprobes$EUR_AF[popprobes$EUR_AF < 0.01]=NA #Give population boundaries

popprobes = popprobes[!is.na(popprobes$EUR_AF),]

GMset.noXY.noPOP = GMset.noXY[which(featureNames(GMset.noXY) %nin% popprobes$PROBE),]

promprobes = read.csv("~/lpersonal/FASD/DNA/annotation/Non-specific-probes-Illumina450k.csv", sep = ",",header = T)

GMset.noXY.noPOP.noPP = GMset.noXY.noPOP[which(featureNames(GMset.noXY.noPOP) %nin% promprobes$TargetID),]

GMset.noXY.noPOP.noPP.noCH = GMset.noXY.noPOP.noPP[grep("ch.", featureNames(GMset.noXY.noPOP.noPP),invert=TRUE),]

Mval = getM(GMset.noXY.noPOP.noPP.noCH)

beta = getBeta(GMset.noXY.noPOP.noPP.noCH) #Extract betas from desired GMset

colnames(beta) = targets$Sample_Name

samplenames = targets$Sample_Name

group = as.factor(targets$Sample_Group) #Set variables

sex = as.factor(targets$Sex)

age = as.numeric(targets$Age)

library(FlowSorted.Blood.450k)

cell = estimateCellCounts(rgSet, compositeCellType = "Blood", processMethod = "preprocessNoob", probeSelect = "auto", cellTypes = c("CD8T","CD4T", "NK","Bcell","Mono","Gran"), returnAll = TRUE, meanPlot = FALSE, verbose = TRUE)

distribution = as.data.frame(cell$counts)

row.names(distribution) = targets$Sample_Name

cd8t = as.numeric(distribution$CD8T)

cd4t = as.numeric(distribution$CD4T)

nk = as.numeric(distribution$NK)

bcell = as.numeric(distribution$Bcell)

mono = as.numeric(distribution$Mono)

gran = as.numeric(distribution$Gran)

design.cor = model.matrix(~0+group+sex+age+cd8t+cd4t+nk+bcell+mono+gran) # Make design matrix

rownames(design.cor) <- targets$Sample_Name

library(limma) #For makeContrasts

cont.matrix = makeContrasts(groupFASD-groupnon_FASD, levels=design.cor) #Set contrasting groups

##Annotation coupling

require("dplyr")

require("tidyverse")

require("data.table")

hg19.gtf = fread("~/lpersonal/FASD/Genome/Homo_sapiens.GRCh37.87.gtf", header = F)

hg19.gtf <- filter(hg19.gtf, V3 == "gene")

hg19.gtf = cbind(hg19.gtf[,1:8], data.frame(do.call('rbind', strsplit(as.character(hg19.gtf$V9), ';', fixed=FALSE))))

hg19.gtf.df = cbind(hg19.gtf[,1],hg19.gtf[,4:5],hg19.gtf[,7],hg19.gtf[,9],hg19.gtf[,11])

names(hg19.gtf.df) = c("chr","start","end","strand","ENS_ID","Gene_Name")

hg19.gtf.df$Gene_Name = gsub("gene_name ", "", hg19.gtf.df$Gene_Name)

hg19.gtf.df$Gene_Name = gsub('"', '', hg19.gtf.df$Gene_Name)

hg19.gtf.df$Gene_Name = trimws(hg19.gtf.df$Gene_Name)

hg19.gtf.df$Gene_Name = as.factor(hg19.gtf.df$Gene_Name)

hg19.gtf.df$ENS_ID = gsub("gene_id ", "", hg19.gtf.df$ENS_ID)

hg19.gtf.df$ENS_ID = gsub('"', '', hg19.gtf.df$ENS_ID)

hg19.gtf.df$ENS_ID = trimws(hg19.gtf.df$ENS_ID)

hg19.gtf.df = hg19.gtf.df %>% filter(str_detect(chr, "GL", negate = TRUE))

hg19.gtf.df = hg19.gtf.df %>% filter(str_detect(chr, "MT", negate = TRUE))

hg19.gtf.df$chr = paste("chr",hg19.gtf.df$chr, sep = "")

hg19.gtf.gr = makeGRangesFromDataFrame(hg19.gtf.df, keep.extra.columns = T, seqnames.field = "chr")

##Ensembl hg38 annotation

hg38.gtf = fread("~/lpersonal/FASD/Genome/Homo_sapiens.GRCh38.97.gtf", header = F)

hg38.gtf <- filter(hg38.gtf, V3 == "gene")

hg38.gtf = cbind(hg38.gtf[,1:8], data.frame(do.call('rbind', strsplit(as.character(hg38.gtf$V9), ';', fixed=FALSE))))

hg38.gtf.df = cbind(hg38.gtf[,1],hg38.gtf[,4:5],hg38.gtf[,7],hg38.gtf[,9],hg38.gtf[,11])

names(hg38.gtf.df) = c("chr","start","end","strand","ENS_ID","Gene_Name")

hg38.gtf.df$Gene_Name = gsub("gene_name ", "", hg38.gtf.df$Gene_Name)

hg38.gtf.df$Gene_Name = gsub('"', '', hg38.gtf.df$Gene_Name)

hg38.gtf.df$Gene_Name = trimws(hg38.gtf.df$Gene_Name)

hg38.gtf.df$Gene_Name = as.factor(hg38.gtf.df$Gene_Name)

hg38.gtf.df$ENS_ID = gsub("gene_id ", "", hg38.gtf.df$ENS_ID)

hg38.gtf.df$ENS_ID = gsub('"', '', hg38.gtf.df$ENS_ID)

hg38.gtf.df$ENS_ID = trimws(hg38.gtf.df$ENS_ID)

hg38.gtf.df = hg38.gtf.df %>% filter(str_detect(chr, "GL", negate = TRUE))

hg38.gtf.df = hg38.gtf.df %>% filter(str_detect(chr, "MT", negate = TRUE))

hg38.gtf.df = hg38.gtf.df %>% filter(str_detect(chr, "KI", negate = TRUE))

hg38.gtf.df$chr = paste("chr",hg38.gtf.df$chr, sep = "")

hg38.gtf.gr = makeGRangesFromDataFrame(hg38.gtf.df, keep.extra.columns = T, seqnames.field = "chr")

##Overlap between EPIC annotation and ensembl gtf annotation

library(plyr)

annotation.hg19.gtf.overlap = as.data.frame(findOverlaps(annotation.gr,hg19.gtf.gr, ignore.strand=TRUE)) #Because the UCSC gene annotation seems not to include strand, for linking Ensembl gene aslo strand is ignored

idx.gtf <- annotation.hg19.gtf.overlap$subjectHits

idx.annotation = annotation.hg19.gtf.overlap$queryHits

annotation.hg19.gtf.gene = cbind(idx.annotation, as.data.frame(hg19.gtf.gr$Gene_Name[idx.gtf],))

annotation.hg19.gtf.gene = unique(annotation.hg19.gtf.gene)

colnames(annotation.hg19.gtf.gene) = c("ID","Gene_Name")

annotation.hg19.gtf.gene.list <- ddply(annotation.hg19.gtf.gene, .(ID), summarize, Gene_Name=paste(Gene_Name,collapse=",")) #library(plyr)

rownames(annotation.hg19.gtf.gene.list) = annotation.hg19.gtf.gene.list$ID

annotation.hg19.gtf.gene.list$ID = NULL

annotation.df = as.data.frame(annotation)

rownames(annotation.df) <- c()

tmp = merge(annotation.df, annotation.hg19.gtf.gene.list, by = 0, all=TRUE,sort = FALSE)

rownames(annotation.df) = annotation.df$Name

tmp = tmp[match(row.names(annotation.df), tmp$Name),]

annotation.df$Gene_Name = tmp$Gene_Name

annotation.df$Gene_Name[is.na(annotation.df$Gene_Name)] <- ""

annotation.df = annotation.df %>% select(1:4, Gene_Name, everything())

annotation.df = annotation.df %>% select(1:5, UCSC_RefGene_Name, everything())

remove("idx.gtf","idx.annotation","tmp")

##DMRcate analysis

require(tidyr)

library(DMRcate)

DMRcate.cpg = cpg.annotate(Mval, datatype = "array", fdr = 0.1, design = design.cor, arraytype = "450K", analysis.type = "differential", what = "M", contrasts = T, cont.matrix = cont.matrix, coef = "groupFASD - groupnon_FASD")

DMRcate.hg19 = extractRanges(dmrcate(DMRcate.cpg, lambda = 1000, C=2),genome="hg19")

DMRcate.hg19 = as.data.frame(DMRcate.hg19)

DMRcate.hg19 = DMRcate.hg19[,1:12]

DMRcate.hg19.gr = makeGRangesFromDataFrame(DMRcate.hg19, keep.extra.columns = T)

DMRcate.hg19.overlap = as.data.frame(findOverlaps(DMRcate.hg19.gr,hg19.gtf.gr, maxgap = 2000))

idx.gene <- DMRcate.hg19.overlap$subjectHits

idx.dmr = DMRcate.hg19.overlap$queryHits

DMRcate.hg19.gene = cbind(DMRcate.hg19.overlap$queryHits, as.data.frame(hg19.gtf.gr$Gene_Name[idx.gene],))

DMRcate.hg19.gene = unique(DMRcate.hg19.gene)

colnames(DMRcate.hg19.gene) = c("ID","Gene_Name")

DMRcate.hg19.gene.list <- ddply(DMRcate.hg19.gene, .(ID), summarize, Gene_Name=paste(Gene_Name,collapse=","))

rownames(DMRcate.hg19.gene.list) = DMRcate.hg19.gene.list$ID

tmp = merge(DMRcate.hg19, DMRcate.hg19.gene.list, by = 0, all=TRUE,sort = FALSE)

DMRcate.hg19 = tmp[2:15][-13][order(tmp$Stouffer),]

DMRcate_unique.hg19 = separate_rows(DMRcate.hg19,Gene_Name, sep = ",", convert = TRUE)

DMRcate_unique.hg19$Gene_Name = trimws(DMRcate_unique.hg19$Gene_Name)

DMRcate_unique.hg19 = na.omit(DMRcate_unique.hg19)

dmrcate_unique.hg19.gr = makeGRangesFromDataFrame(DMRcate_unique.hg19, keep.extra.columns = T)

remove("idx.gene","idx.dmr","tmp")

##lmFit analysis

lmfit.cor = lmFit(beta, design.cor, method="ls") #Lineair model correction, set method as desired (or use ls)

lmfit.cor.contrast = contrasts.fit(lmfit.cor,cont.matrix)

lmfit.cor.contrast.eB = eBayes(lmfit.cor.contrast) #Bayes to moderate the standard deviations between genes

lmfit.cor.contrast.eB.fdr = topTable(lmfit.cor.contrast.eB, n=nrow(beta), adjust.method="fdr")

lmfit.cor.contrast.eB.BH = topTable(lmfit.cor.contrast.eB, n=nrow(beta), adjust.method="BH")

lmfit = cbind(lmfit.cor.contrast.eB.fdr, lmfit.cor.contrast.eB.BH[5])

colnames(lmfit)[7] = "BH"

beta.df = as.data.frame(beta)

colnames(beta.df) = targets$Sample_Group

beta.df$Delta = c(rowMeans(beta.df[,colnames(beta.df)=="FASD"]) - rowMeans(beta.df[,colnames(beta.df)=="non_FASD"])) #Calculate Delta

tmp = data.frame(beta.df[match(row.names(lmfit), row.names(beta.df)),])

lmfit = cbind(lmfit,tmp$Delta)

colnames(lmfit)[colnames(lmfit)=="tmp$Delta"] = "Delta"

tmp = annotation.df[match(row.names(lmfit), row.names(annotation.df)),]

lmfit = cbind(lmfit,tmp)

tmp = data.frame(lmfit[match(row.names(beta.df), row.names(lmfit)),])

beta.df=cbind(as.data.frame(beta.df),tmp$P.Value, tmp$adj.P.Val,tmp$chr,tmp$pos)

colnames(beta.df) = as.character(c(targets$Sample_Name,"Delta","P.Value","q.value","chr","pos"))

remove("tmp")

##RNA data analysis

require(DESeq2)

library(ggrepel)

gcounts <- data.frame(fread("~/lpersonal/FASD/RNA/103166/output/counts/counts.txt",sep = "\t"), row.names=1)

targets$GS_ID=gsub('-', '.', targets$GS_ID)

gcounts = gcounts %>% select(ends_with(paste(targets$GS_ID,".bam", sep = "")))

colnames(gcounts) = targets$Sample_Name

##Link multiple reads with same gene

tmp = gcounts

tmp$ENS_ID = rownames(tmp)

tmp2 = right_join(hg38.gtf.df[,c("ENS_ID", "Gene_Name")], tmp, by = c("ENS_ID" = "ENS_ID"))

tmp3 = as.data.frame(tmp2 %>% mutate(Gene_Name = coalesce(Gene_Name,ENS_ID)))

tmp3 = tmp3[2:ncol(tmp3)]

tmp3 <- tmp3[which(!rowSums(tmp3 == 0)>ncol(gcounts)*0.5),]

tmp4 = data.frame()

i=2

repeat{ # Start repeat-loop

tmp5 <- aggregate(tmp3[[i]] ~ Gene_Name, tmp3, sum)

if (i < 3){tmp4 = tmp5} else{

tmp4 = cbind(tmp4,tmp5[2])

}

remove(tmp5)

i <- i + 1

if(i > length(samplenames)+1) {

break

}

}

colnames(tmp4) = colnames(tmp3)

row.names(tmp4) = tmp4$Gene_Name

gcounts_clean = tmp4[2:length(tmp4)]

RNA = as.matrix(tmp4[,2:(1+ncol(gcounts))])

remove("i","tmp","tmp2","tmp3","tmp4")

dds = DESeqDataSetFromMatrix(countData = gcounts_clean, colData = targets, design = ~Sex+Age+CD8T+CD4T+NK+Bcell+Mono+Gran+Sample_Group)

dds.deseq <- DESeq(dds, fitType="local")

ddsresults = as.data.frame(results(dds.deseq, contrast=c("Sample_Group","FASD","non_FASD")))

ddsresults <- ddsresults[!is.na(ddsresults$padj),]

ddsresults$ENS_ID = row.names(ddsresults)

ddsresults <- right_join(hg38.gtf.df[,c("ENS_ID", "Gene_Name")], ddsresults, by = c("Gene_Name" = "ENS_ID"))

ddsresults.df = cbind(ddsresults,hg38.gtf.df$start[match(ddsresults$Gene_Name,hg38.gtf.df$Gene_Name)])

ddsresults.df = cbind(ddsresults.df,hg38.gtf.df$end[match(ddsresults.df$Gene_Name,hg38.gtf.df$Gene_Name)])

ddsresults.df = cbind(ddsresults.df,hg38.gtf.df$chr[match(ddsresults.df$Gene_Name,hg38.gtf.df$Gene_Name)])

names(ddsresults.df)[9:11] = c("start", "end","chr")

ddsresults.df = ddsresults.df[complete.cases(ddsresults.df$start), ]

ddsresults.df <- as.data.frame(ddsresults.df[order(ddsresults.df$pvalue),])

row.names(ddsresults.df) = ddsresults.df$ENS_ID

##Annotation and eQTM analsye

require("NDlib")

require(eqtm)

eqtm.annot.gr = makeGRangesFromDataFrame(getAnnotation(GMset.noXY.noPOP.noPP.noCH), start.field = "pos", end.field = "pos", keep.extra.columns = T)

eqtm.hg19 <- eqtm(dmrs_gr = dmrcate_unique.hg19.gr,

gene_col = "Gene_Name", , gene_col = "Gene_Name", meth_data = beta, expr_data = RNA, meth_anno_gr = eqtm.annot.gr, cor_method = "pearson", aggregation_method = "median", alternative = "two.sided", N = 100000, iseed = 531235, ncores = 16)

eqtms.hg19 <- data.frame(rowRanges(eqtm.hg19))

eqtms.hg19 <- eqtms.hg19[order(eqtms.hg19$nCpGs, decreasing = T),]

eqtms.topcor.hg19 <- eqtm::topcor(eqtm.hg19, sort.by = "pval")

##Overlap DMRcate and expression data with rightjoin script, links gene name

overlap = right_join(ddsresults.df, DMRcate_unique.hg19[,c("Gene_Name","Stouffer")], by = c("Gene_Name" = "Gene_Name"))

overlap = overlap[complete.cases(overlap$pvalue), ]

overlap.unsig = subset(overlap, overlap$pvalue > 0.05 & overlap$Stouffer > 0.05)

overlap.sig.expr = subset(overlap, overlap$pvalue < 0.05 & overlap$Stouffer > 0.05)

overlap.sig.meth = subset(overlap, overlap$Stouffer < 0.05 & overlap$pvalue > 0.05)

overlap.sig.expr.meth = subset(overlap, overlap$pvalue < 0.05 & overlap$Stouffer < 0.05)

###########################################################################################################################################

sessionInfo()
