# Supplementary material for: Expression Quantitative Trait Methylation Analysis Identifies Whole Blood Molecular Footprint in Fetal Alcohol Spectrum Disorder (FASD)
Source: Int J Mol Sci. 2023 Apr 1;24(7):6601. doi: 10.3390/ijms24076601 (PMC10095438; doi:10.3390/ijms24076601)
Supplement: Supplementary file 1 [file ijms-24-06601-s001.zip › legend.docx]

Supplementary Table S1 (Excel File). Supplementary Figure S1. Correlation of PCs with batch effects and metadata. (A) Pearson correlation (y-axis) of the first eight PCs (x-axis) with metadata: group (FASD or control), Sex, Age, Array (position), and slide. (B) Pearson correlation (y-axis) of the first eight pPCs (x-axis) with estimated (Housman’s method) relative blood cell type counts of CD8+, CD4+, natural killer cell, B-cell, granulocyte, and monocyte, respectively. (C) PCA of DNAm data. (D) Variance explained by PCs. Supplementary Figure S2. Volcano plot of DMPs (adjusted for sex, age, and cell types). X-axis represents mean effect size (delta *β*-value) and y-axis represents –log10(*p*-value). Orange dots indicate probes with FDR >0.05 and blue dots indicate genome-wide significant CpGs (FDR < 0.05). Supplementary Figure S3. PCA of RNA-seq data. Supplementary Figure S4. WGCNA module–trait correlations. Correlation values are given with *p*-values within parentheses. Module brown shows a significant negative correlation with FASD. Supplementary Figure S5. Overlap of DMRs with RNA-seq results. Overlap of top FASD DMRs with RNA-seq. X-axis represents -log10(Stouffer’s coefficient) of DMRs and Y-axis represents –log10(*p-*value) of genes from the differential gene expression analysis. Dot annotation: red, insignificant; blue, gene with nominal *p*-value < 0.05; green, DMR genome-wide significant; purple, DMR genome-wide significant and gene with nominal *p*-value < 0.05. Supplementary File S1. Scripts
