# Supplementary figures and images for: Expression Quantitative Trait Methylation Analysis Identifies Whole Blood Molecular Footprint in Fetal Alcohol Spectrum Disorder (FASD)
Source: Int J Mol Sci. 2023 Apr 1;24(7):6601. doi: 10.3390/ijms24076601 (PMC10095438; doi:10.3390/ijms24076601)

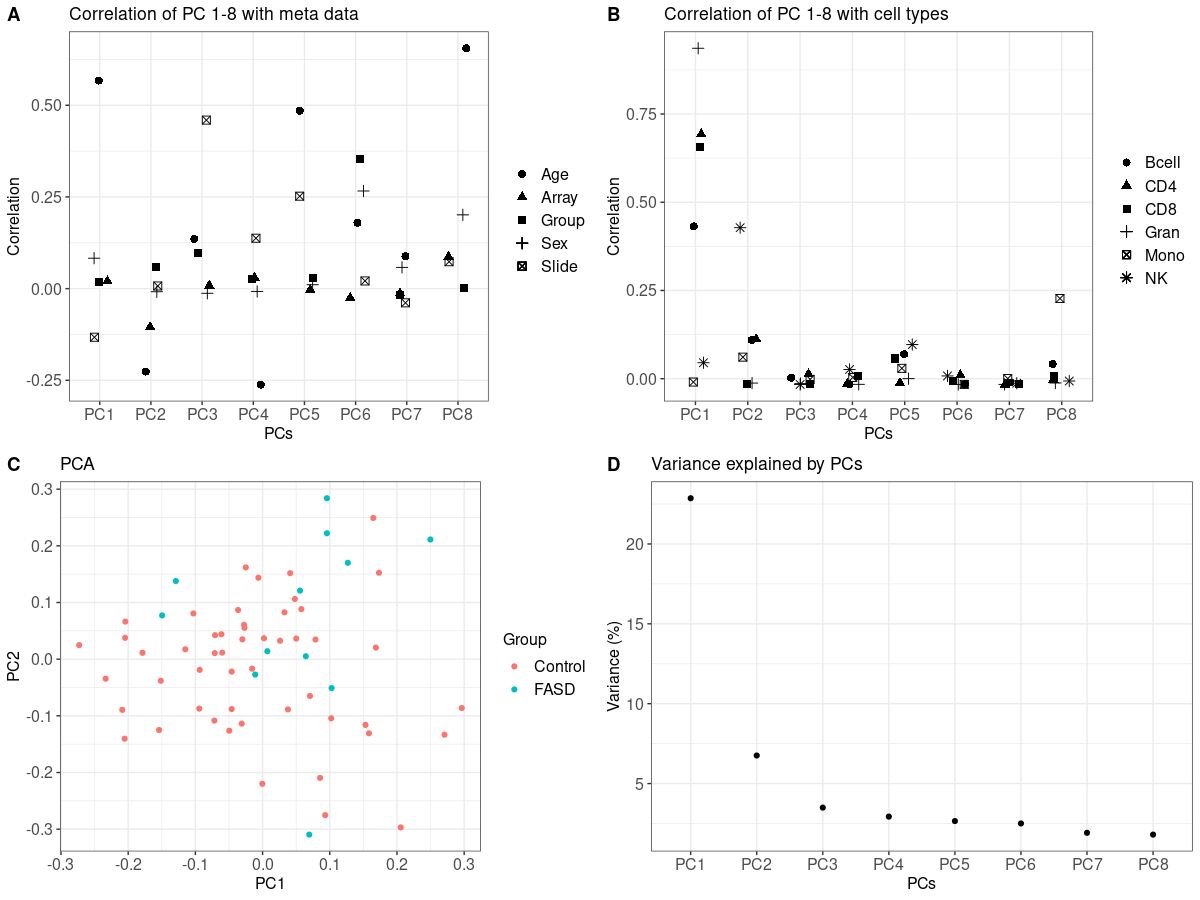

Supplement: Supplementary file 1 [file ijms-24-06601-s001.zip › FASD_SupplementaryFigureS1.tiff]

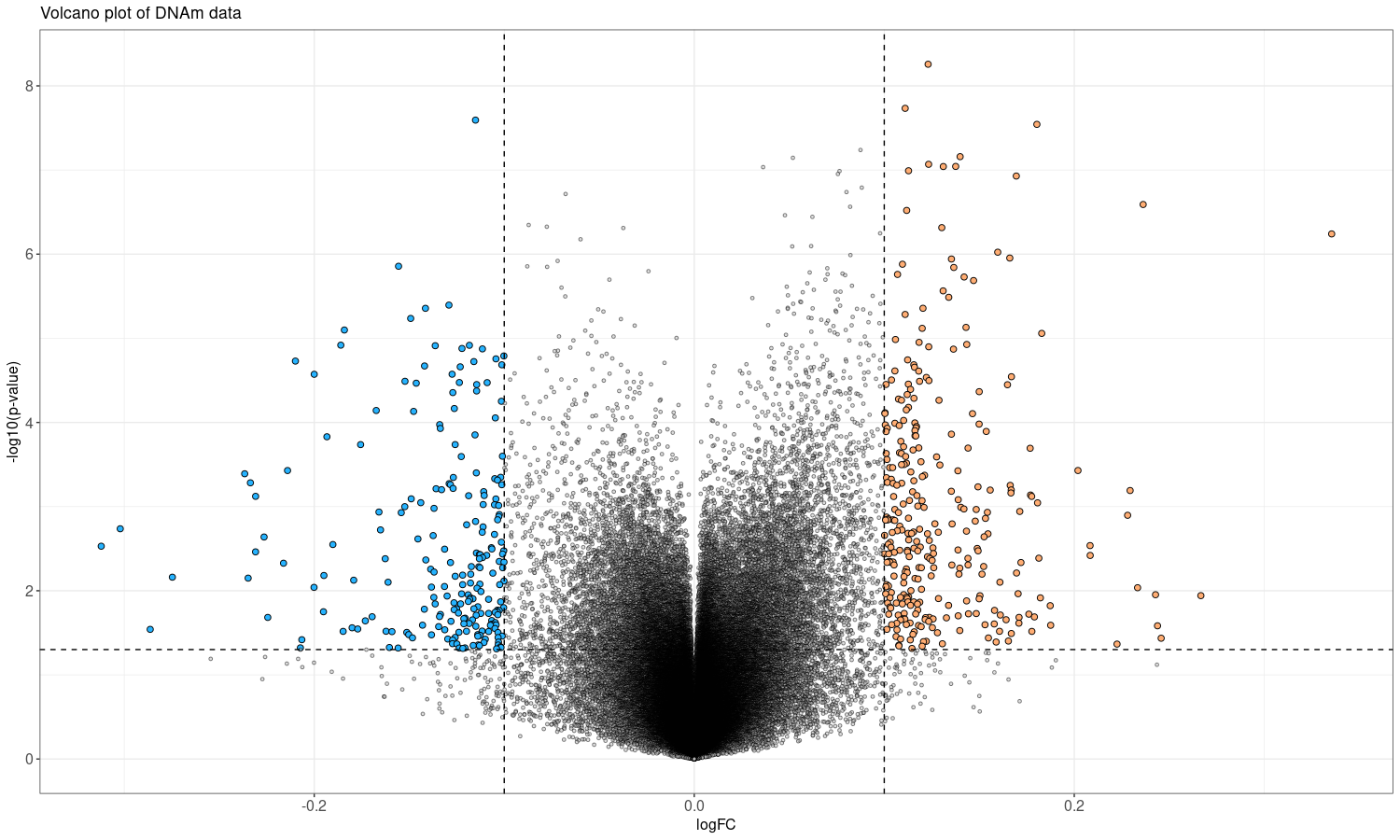

Supplement: Supplementary file 1 [file ijms-24-06601-s001.zip › FASD_SupplementaryFigureS2.tiff]

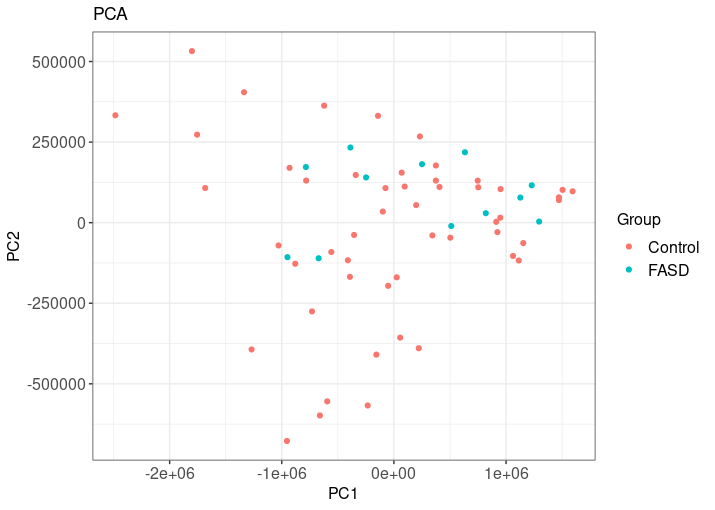

Supplement: Supplementary file 1 [file ijms-24-06601-s001.zip › FASD_SupplementaryFigureS3.tiff]

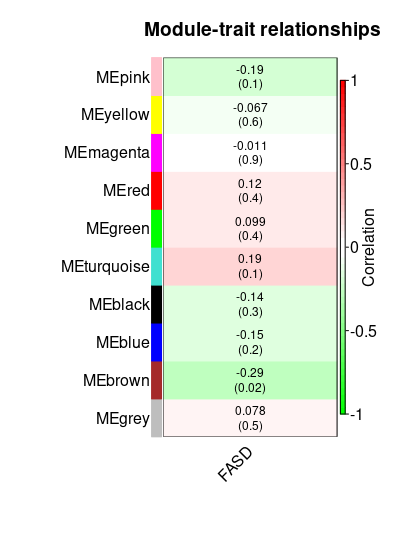

Supplement: Supplementary file 1 [file ijms-24-06601-s001.zip › FASD_SupplementaryFigureS4.tiff]

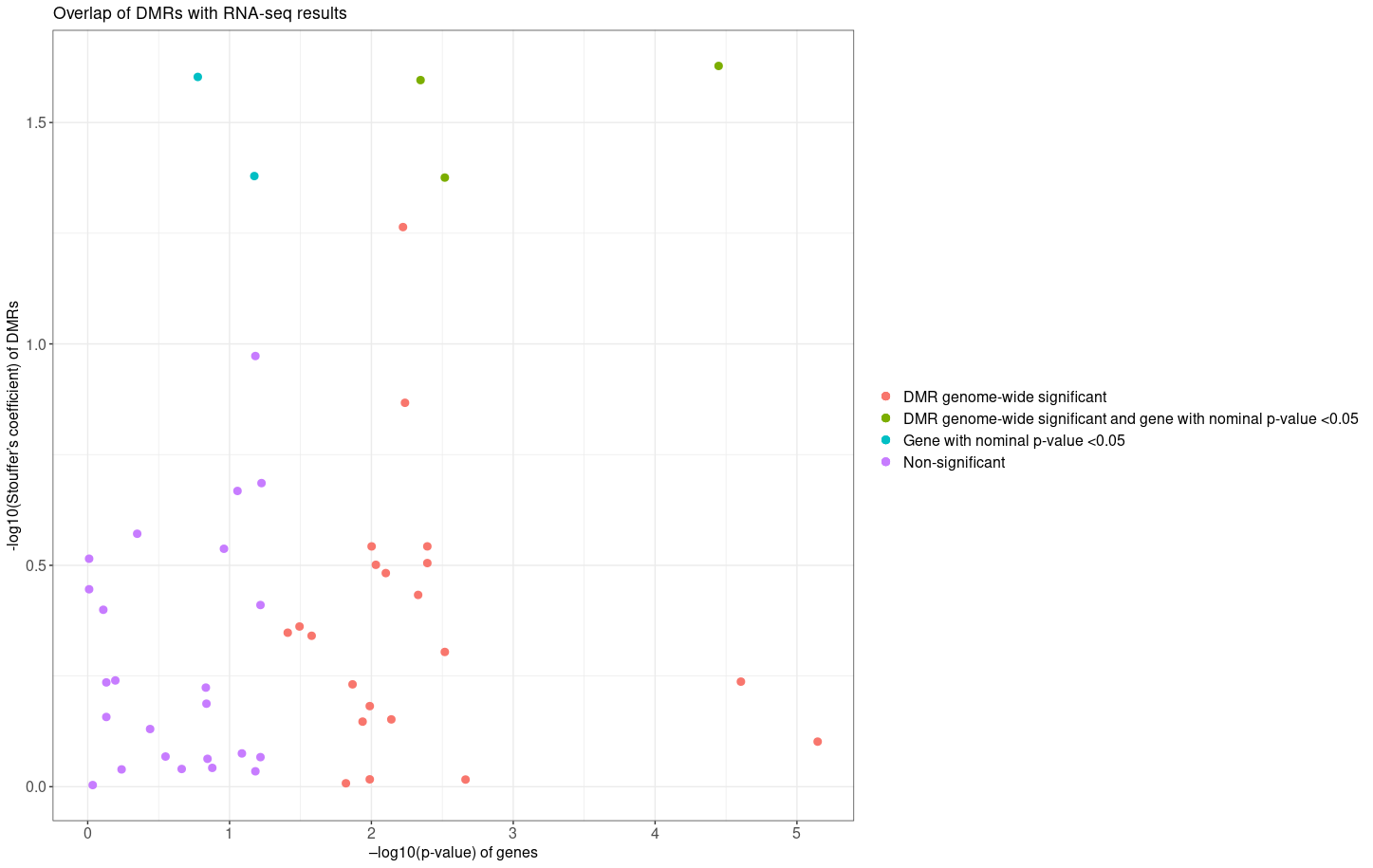

Supplement: Supplementary file 1 [file ijms-24-06601-s001.zip › FASD_SupplementaryFigureS5.tiff]
